# Environmental Exosomes/Small Extracellular Vesicles: Evidence of Extracellular RNA Release by Aquatic Organisms

**DOI:** 10.1007/s10126-026-10618-1

**Published:** 2026-04-25

**Authors:** Ryo Yonezawa, Lingxin Meng, Naoki Hashimoto, Ibuki Igarashi, Satoshi Kimura, Nina Yasuda, Susumu Mitsuyama, Takanori Kobayashi, Kazutoshi Yoshitake, Shigeharu Kinoshita, Nahoko Bailey-Kobayashi, Kaoru Maeyama, Kiyohito Nagai, Shugo Watabe, Tetsuhiko Yoshida, Shuichi Asakawa

**Affiliations:** 1 Laboratory of Aquatic Molecular Biology and Biotechnology, Department of Aquatic Bioscience, Graduate School of Agricultural and Life Science, The University of Tokyo, Bunkyo, Tokyo, 113-8657 Japan; 2 Signal Peptidome Research Laboratory, Department of Aquatic Bioscience, Graduate School of Agricultural and Life Sciences, The University of Tokyo, Bunkyo, Tokyo, 113-8657 Japan; 3 Laboratory of Aquatic Conservation, Department of Ecosystem Studies, Graduate School of Agricultural and Life Science, The University of Tokyo, Bunkyo, Tokyo, 113-8657 Japan; 4 Technology Advancement Center, Graduate School of Agricultural and Life Sciences, The University of Tokyo, Bunkyo-ku, Tokyo, 113-8657 Japan; 5 College of Bioresource Sciences, Nihon University, Kanagawa, 252-0880 Japan; 6 School of Marine Biosciences, Kitasato University, Minami-ku, Sagamihara, Kanagawa 252-0313 Japan; 7 Institute for Advanced Sciences, TOAGOSEI CO., LTD, Tsukuba, Ibaraki 300-2611 Japan; 8 Mikimoto Pearl Research Institute, K.MIKIMOTO & CO., LTD, Hazako, Hamajima, Shima, Mie 923-74, 517-0403 Japan; 9 Mikimoto Pharmaceutical CO., LTD, Kurose, Ise, Mie 1425, 516-8581 Japan

**Keywords:** Environmental exosomes/small extracellular vesicles (eExosome/esEVs), Exosome, Small extracellular vesicle, Environmental DNA/RNA (eDNA/eRNA) analysis, Small RNA

## Abstract

Aquatic organisms continuously interact with surrounding water, yet whether they release extracellular vesicles into this vast medium remains unknown. We hypothesized that pearl oysters (*Pinctada fucata*) release exosomes/small extracellular vesicles (sEVs) into the aquatic environment. To this end, we collected exosome/sEV-sized components by ultrafiltration from tank water and open-sea culture areas. Microscopy revealed abundant vesicles consistent with exosome/sEV size, and small RNA sequencing identified pearl oyster-specific PIWI-interacting RNAs (piRNAs) that matched sequences previously detected in hemolymph exosomes. These findings demonstrate that pearl oysters actively released exosomes containing species-specific nucleic acids into surrounding water, effectively protecting the RNA from rapid degradation. We propose referring to these vesicles as environmental exosomes/environmental sEVs (eExosomes/esEVs). These findings suggest that aquatic exosomes/sEVs serve as carriers of RNA and may contribute to inter-organismal communication networks. Beyond their functional role, eExosomes/esEVs also hold promise as highly stable, novel targets for environmental DNA/RNA (eDNA/eRNA) analysis, offering new opportunities for ecological monitoring and biodiversity research.

**Supplementary Information:**

The online version contains supplementary material available at 10.1007/s10126-026-10618-1.

## Introduction

Aquatic organisms continuously exchange biological components with their surrounding waters. These exchanges can profoundly influence ecological interactions. We hypothesized that, in addition to soluble biomolecules, exosomes and small extracellular vesicles (sEVs) may also be secreted into aquatic environments. These vesicles could act as stable carriers of biological information not only within organisms (as they do in terrestrial species, transferring information among tissues and organs), but also between organisms. Exosomes and sEVs are lipid bilayer vesicles typically 50–200 nm in diameter (Pegtel and Gould 2019; Gurung et al. 2021; Nazri et al. 2023; Ważny et al. 2024; Gabaran et al. 2025; Zhou et al. 2025) known to encapsulate nucleic acids, proteins, and other bioactive molecules (Meldolesi 2018; Pegtel and Gould 2019; Kalluri and LeBleu 2020; Negahdaripour et al. 2021) and mediate a wide range of intercellular communication processes (Valadi et al. 2007; Meldolesi 2018; Pegtel and Gould 2019; Gurung et al. 2021). In terrestrial and biomedical research, extensive studies have revealed their critical roles in physiological regulation, disease mechanisms, and cell-to-cell signaling (Kim et al. 2015; Zhao et al. 2020; Dad et al. 2021; Moros et al. 2021; Chen and Li 2025). However, investigations into the roles of exosomes/sEVs in aquatic organisms remain limited. Most prior work has focused on intracellular or organismal functions, while their potential release into the external aquatic environment and possible contributions to inter-organismal communication remain essentially unexplored.

Extracellular vesicles, such as exosomes, possess a lipid bilayer that protects their enclosed nucleic acids and proteins from enzymatic degradation (Koga et al. 2011; Chen et al. 2024), potentially enabling their persistence in aquatic environments. If released into surrounding waters, these vesicles could serve as vehicles for the transfer of functional molecules between individuals, thus contributing to ecological communication networks. Our previous studies on the Akoya pearl oyster (*Pinctada fucata*) have demonstrated that hemolymph-derived exosomes are enriched in small RNAs, particularly PIWI-interacting RNAs (piRNAs; Huang et al. 2022), which are expressed in various somatic tissues of invertebrates (Huang et al. 2019, 2021). Notably, invertebrate piRNAs appear to serve broader functions, potentially resembling those of microRNAs (miRNAs; Huang et al. 2021). Empirical evidence supports this possibility; for instance, plant-derived miRNAs have been shown to regulate insect gene expression (Shi et al. 2024), and algae-derived exosomes containing miRNAs have been detected in *P. fucata* (Zheng et al. 2022). Based on these integrated biological foundations, we conceived the idea that aquatic organisms might continuously release exosomes/sEVs into the surrounding environment, functioning as highly stable, transmissive carriers for small RNA-mediated inter-organismal communication.

In recent years, environmental nucleic acids, especially eRNA in aquatic ecosystems, have emerged as powerful tools for assessing biodiversity and conducting ecological monitoring (Minamoto et al. 2021; Veilleux et al. 2021; Yates et al. 2021; Glover et al. 2025). Because RNA reflects the active physiological state of living organisms, eRNA applications have expanded from precise ecological monitoring (Miyata et al. 2021, 2025) to the detection of functional and stress responses via environmental transcriptomics (Hiki et al. 2023; Hechler et al. 2025; Hiki and Jo 2025). However, eRNA analysis faces significant technical challenges and limitations, such as the overwhelming dominance of non-target microbial RNA (Hiki et al. 2023; Hechler et al. 2025; Hiki and Jo 2025) and inherently rapid degradation (Wang et al. 2025). Unlike eDNA, free eRNA is highly susceptible to rapid degradation depending on environmental factors such as temperature and pH (Kagzi et al. 2022; Jo et al. 2023), with the half-lives of some specific transcripts estimated to be as short as a few tens of minutes (Aminaka et al. 2025). Consequently, while controlled tank and laboratory experiments have provided valuable insights into eRNA dynamics, standardized protocols and successful applications in complex natural field environments remain scarce (Bunholi et al. 2023). While some researchers in the eRNA field have recently begun to speculate that eRNA persistence might depend on protection by biological structures like extracellular vesicles (Jo et al. 2022), direct empirical evidence from natural environments has been lacking. This lack of evidence is largely due to the technical difficulty of recovering and concentrating exosomes/sEVs from highly diluted environmental water, as no standardized isolation protocols exist for field samples.

Therefore, driven by our biological hypothesis regarding small RNA dynamics and vesicle-mediated protection, in this study, we aimed to provide the first direct evidence that Akoya pearl oysters release exosomes and sEVs into surrounding seawater as highly stable alternative carriers, by combining microscopy-based characterization with small RNA sequencing. By validating the presence of these carriers in the natural environment, we sought to uncover a previously unrecognized mechanism of molecular exchange in aquatic ecosystems.

## Materials and Methods

### Biological Materials

**Fig. 1:**
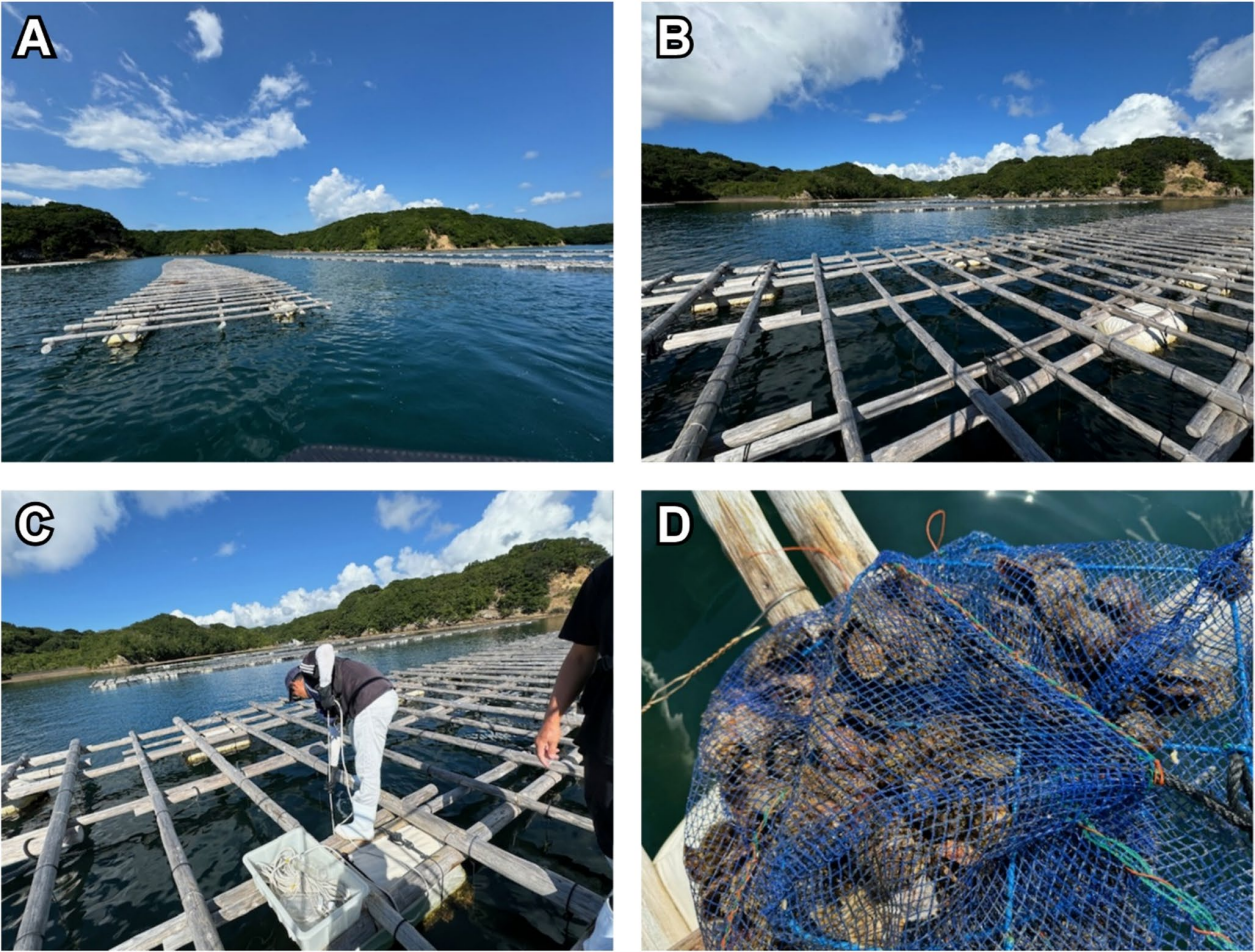
Sampling sites and collection procedures in the aquaculture area. (**A**): The raft in Ago Bay, Shima, Mie prefecture, where Akoya pearl oysters are cultured. (**B**): Raft used for farming Akoya pearl oysters. (**C**): Water collection procedure. (**D**): Suspended net cage for Akoya pearl oyster marine farming

In September 2023, a tank containing Akoya pearl oysters (summer-harvested seed bivalves, 300 individuals in a 30-L tank with 20 L of filtered seawater) was maintained for 5 days under static water conditions without feeding. As the initial findings were derived from closed-environment tank water, we extended our investigation to open-water conditions conducted concurrently. In September 2024, 2 L of seawater was collected in the morning and divided into two 1-L replicates (hereafter referred to as AM1 and AM2). Similarly, another 2 L was collected in the evening and divided into two 1-L replicates (PM1 and PM2), from the Akoya pearl oyster aquaculture area (34°17’41"N 136°48’09"E) in Ago Bay, Mie Prefecture, Japan (Fig. 1). After collection, the water samples were stored at 4 °C for 1 day and transported to the University of Tokyo, Tokyo, Japan, via refrigerated delivery at 4 °C. Samples were processed immediately upon arrival. This research was approved by the Animal Experiment Ethics Committee of the Graduate School of Agricultural and Life Science, The University of Tokyo (Accession No. P21-103).

### Environmental Exosome/sEV Isolation and Identification

**Fig. 2:**
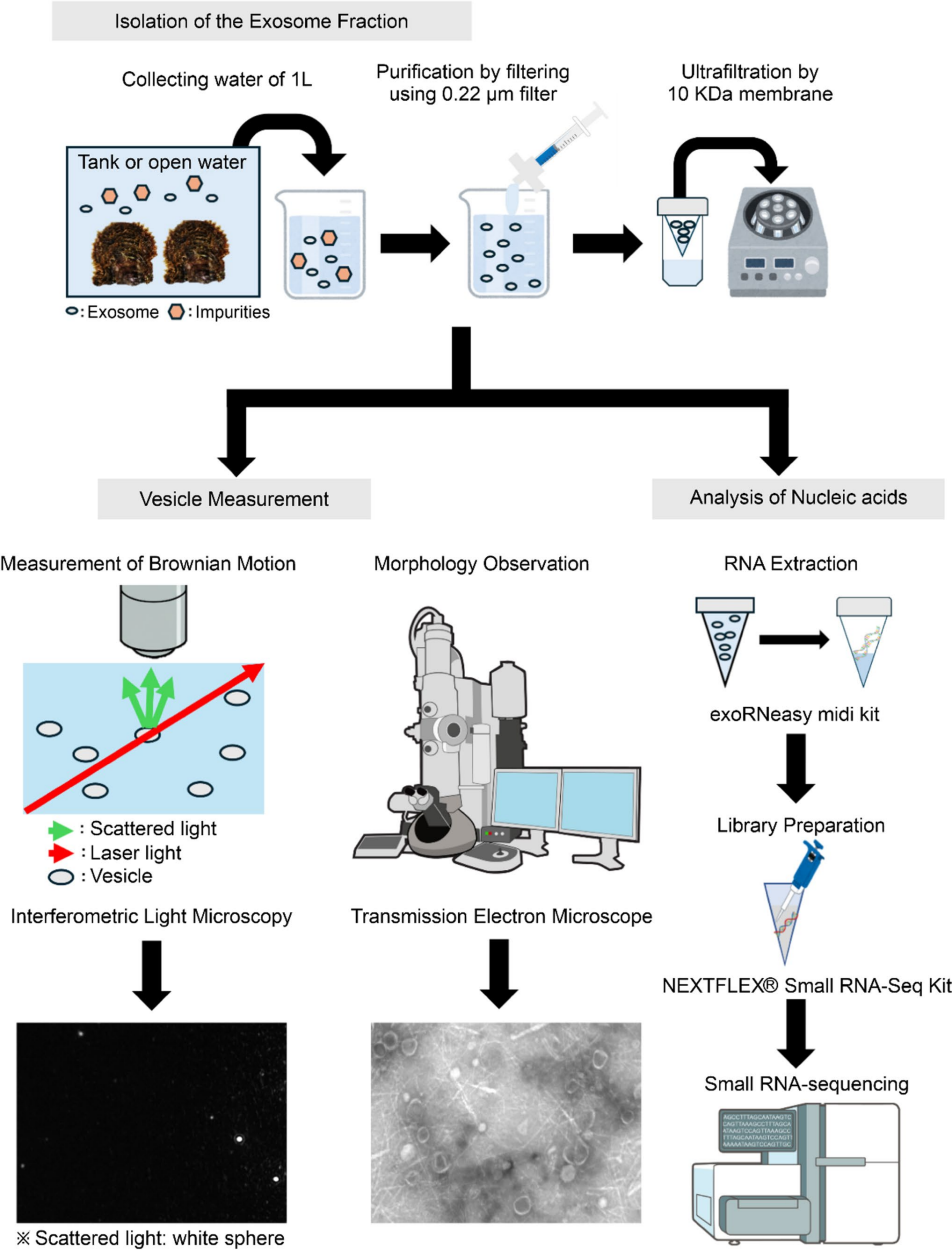
Experimental design and analysis workflow performed in this study

As shown in Fig. 2, exosomes/sEVs were recovered by modifying an exosome purification protocol originally designed for culture supernatants (Chen et al. 2022). To isolate vesicles within the exosome/sEV size range (50–200 nm), seawater was first filtered through a 20-µm mesh to remove large particulates. The filter was then passed through a 0.22 μm Stericup-GP filter (Merck Millipore, MA, USA) to eliminate residual smaller particulates. The final filtrate, containing vesicles smaller than 220 nm, was subjected to ultrafiltration.

Ultrafiltration was performed on 1 L of sample using Centricon Plus-70 10 kDa Ultracel-PL units (Merck Millipore) at 3,500 × g for over 25 min (Centrifuge; Model 6200; rotor: SF-2504 S; KUBOTA, Tokyo, Japan). After removing the flow-through, the retained fraction was replenished with the remaining sample and repeatedly concentrated. The final concentrate was replaced with phosphate buffered saline (PBS) and stored at −80 °C until further use. For the aquaculture open-water samples, vesicle concentration and particle size distribution were analyzed using interferometric light microscopy (Videodrop), which measures Brownian motion (Sausset et al. 2023). For this analysis, 100 µL samples from the AM and PM collections were sent to Meiwafosis (Tokyo, Japan). Vesicle counts per liter were estimated based on these measurements.

For transmission electron microscopy (TEM), samples from both the rearing tank and aquaculture area were prepared. Exosome/sEV fractions were further purified via ultracentrifugation at 100,000 × g for 70 min at 4 °C, supernatant was removed, and the pellet was resuspended in PBS. Ultracentrifugation was performed using an Optima MAX-TL ultracentrifuge (Beckman Coulter, CA, USA) with a TLA-100.3 rotor (Beckman Coulter). Samples were applied to self-made carbon coated copper grids (200 mesh), allowed to adsorb for less than 5 min, and negatively stained with 2% uranyl acetate (Bio-Rad, CA, USA) for approximately 30 s. The grids were then visualized using a transmission electron microscope (JEM-1400plus; JEOL, Tokyo, Japan) operated at an accelerating voltage of 120 kV.

### RNA Extraction, Library Construction, and Small RNA Sequencing

Following the methodology described by Pan et al. (2025), small RNA was extracted from each exosome/sEV fraction obtained from tank water (250 µL) and open water (300 µL) utilizing the exoRNeasy midi kit (Qiagen), in accordance with the manufacturer’s protocol. The quantity and quality of the extracted small RNA were evaluated using the Qubit microRNA kit with the Qubit 2.0 fluorometer (Thermo Fisher Scientific, MA, USA) and the Agilent Small RNA kit with the Agilent 2100 Bioanalyzer (Agilent Technologies, CA, USA). Library construction was carried out using the NEXTFLEX^®^ Small RNA-Seq Kit v4 (Revvity, MA, USA), following the manufacturer’s instructions. For the evening samples, the cDNA amplification step was modified to increase the number of PCR cycles to 32 in order to obtain sufficient cDNA yields. Final library quality was evaluated using the High-Sensitivity D1000 ScreenTape Kit on the Agilent 2200 TapeStation. High-quality libraries were sequenced using the DNBSEQ-G400 platform (BGI) with 100 bp paired-end reads by BGI JAPAN (Hyogo, Japan).

### Sequencing Data Analysis

Following the method described by Meng et al. (2025), we first trimmed adapter sequences and removed low-quality reads. Reads outside the 18–40 nt range were filtered using TrimGalore (https://github.com/FelixKrueger/TrimGalore). The remaining reads were mapped to the *P. fucata* reference genome (Takeuchi et al. 2022) using Bowtie (Langmead et al. 2009) with zero mismatches (bowtie -f -v 0 -a --al) to analyze expression and distribution across the genome. To improve alignment efficiency, reference haplotypes, versions 4.1 A (reference) and 4.1B (alternative), were used. To classify and annotate small RNAs, mapped reads were compared against Mollusca sequences in the miRBase v22.1 database (Kozomara et al. 2019) allowing for one mismatch, using Bowtie (bowtie -v 1 -a --best --strata). The reads were also annotated against the Rfam 14.10 database (Kalvari et al. 2020) to identify other small RNA types (e.g., rRNAs, tRNAs), using Bowtie with no mismatches (bowtie -v 0 -a --best --strata). GNU Awk v4.0.2 (https://www.gnu.org/software/gawk/) was used to extract the sequences.

Subsequently, a custom Perl script (casify_rna.pl; Supplementary data 1), developed with the assistance of ChatGPT (GPT-5; OpenAI) based on reference (Zhang et al. 2023), was used to classify RNA and output FASTA sequences (perl classify_rna.pl -fa < fasta> -rfam < rfam_file> -mirna < miRNA.bwt> -ncrna < ncRNA.bwt> -outpre < output_prefix>). The authors reviewed and validated the generated code to ensure its accuracy. During annotation, sequences of unexpected lengths were observed among the classified miRNAs. Canonical miRNA sequences (20–24 nt) were extracted using seqkit (Shen et al. 2016); all others were designated as “unknown”. As Rfam lacks sequences specific to *P. fucata*, previously published sequences (28 S rRNA: AB214477, 18 S rRNA: AB214462, 5.8 S rRNA: AB205102, ITS1: AB214218, ITS2: AB214265, rRNA intergenic spacer: AB214291 and AB214307) were used to recover unannotated rRNAs from the unknown category via local BLAST (BLAST 2.16.0+). Tandem repeats were then removed from the unknown sequences using MISA (Beier et al. 2017) and TRF (Benson 1999). Sequences 25–32 nt in length were extracted and designated as predicted piRNAs, following the criteria in Huang et al. (2022). In model organisms, piRNAs frequently show a 1U bias, with uridine at the first position (Kawaoka et al. 2011; Izumi et al. 2013; Stein et al. 2019). Accordingly, we analyzed the nucleotide composition at the first base of the predicted piRNAs, evaluating both the diversity and frequency of occurrence. Finally, we used piRNAs derived from *P. fucata* hemolymph exosomes (Huang et al. 2022) as a reference database to identify perfectly matching sequences via BLASTn (option: -task blastn-short).

Additionally, for the Tank and aquaculture site (AM1) sample, which yielded the highest number of sequencing reads, adapter trimming was performed using fastp (Chen et al. 2018). The resulting paired-end reads were mapped to the *P. fucata* reference genome using BWA-MEM (Li 2013), and alignment files were processed with SAMtools (Li et al. 2009) to generate high-quality mapped reads. To account for potential splicing, alignments were also performed using HISAT2 (Kim et al. 2019), followed by transcript assembly with StringTie (Pertea et al. 2015). A merged GTF file, generated from both genome versions 4.1 A and 4.1B, was used to detect transcripts from both haplotypes. CDS and peptide sequences were predicted from the assembled transcripts using TransDecoder (Haas et al. 2013). Functional annotation was conducted via BLASTx and BLASTp searches against the Swiss-Prot database (downloaded December, 2023), and comprehensive annotations were generated using Trinotate (Bryant et al. 2017), which incorporated protein domain and functional annotation data.

## Results

### Identification of Exosomes/sEVs from Environmental Seawater

**Fig. 3:**
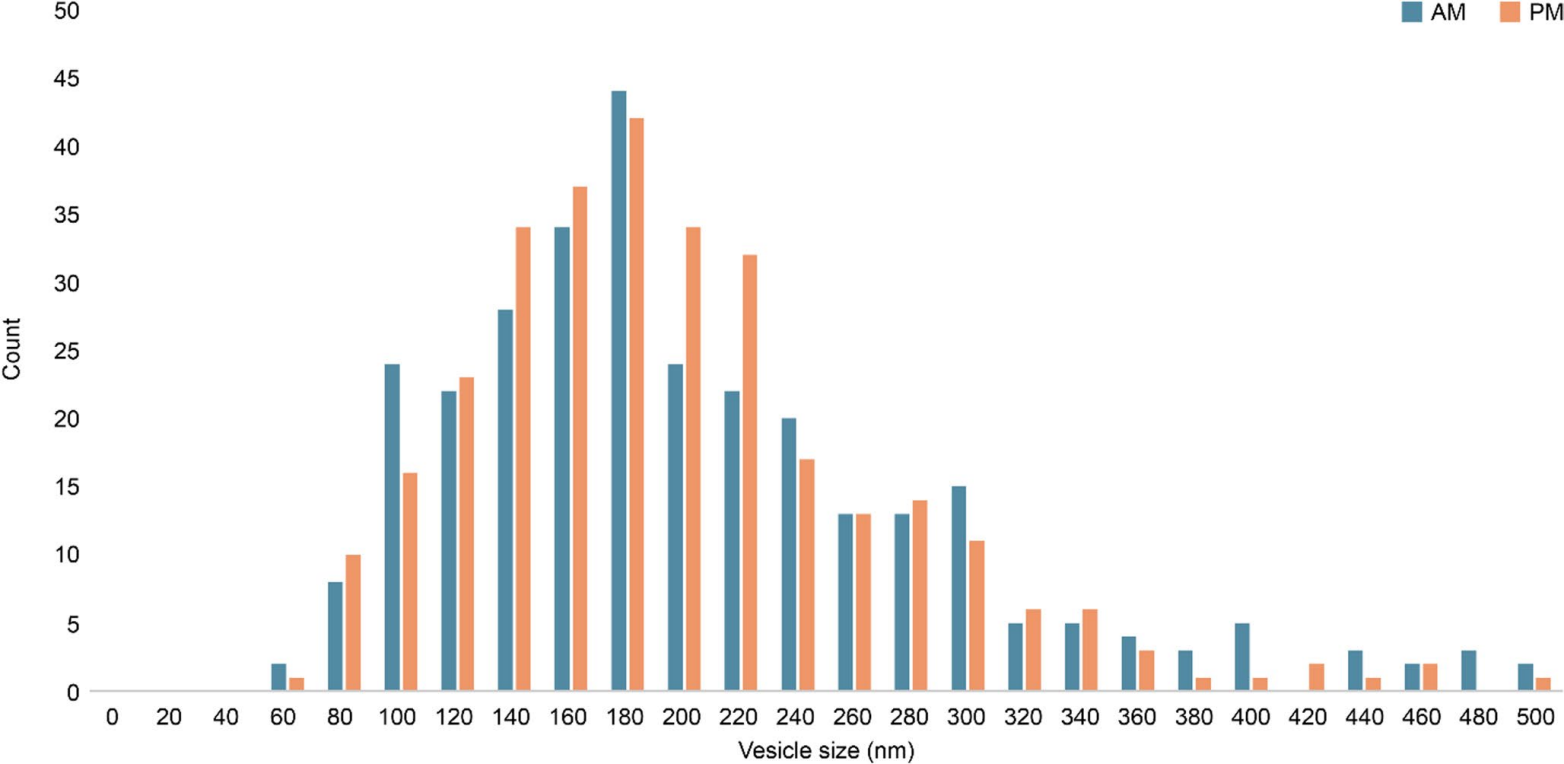
Exosome/sEV fraction identification using interferometric light microscopy of Videodrop. Vesicle size distribution of exosomes/sEVs isolated from open-water samples collected in the morning (AM) and evening (PM)

**Fig. 4:**
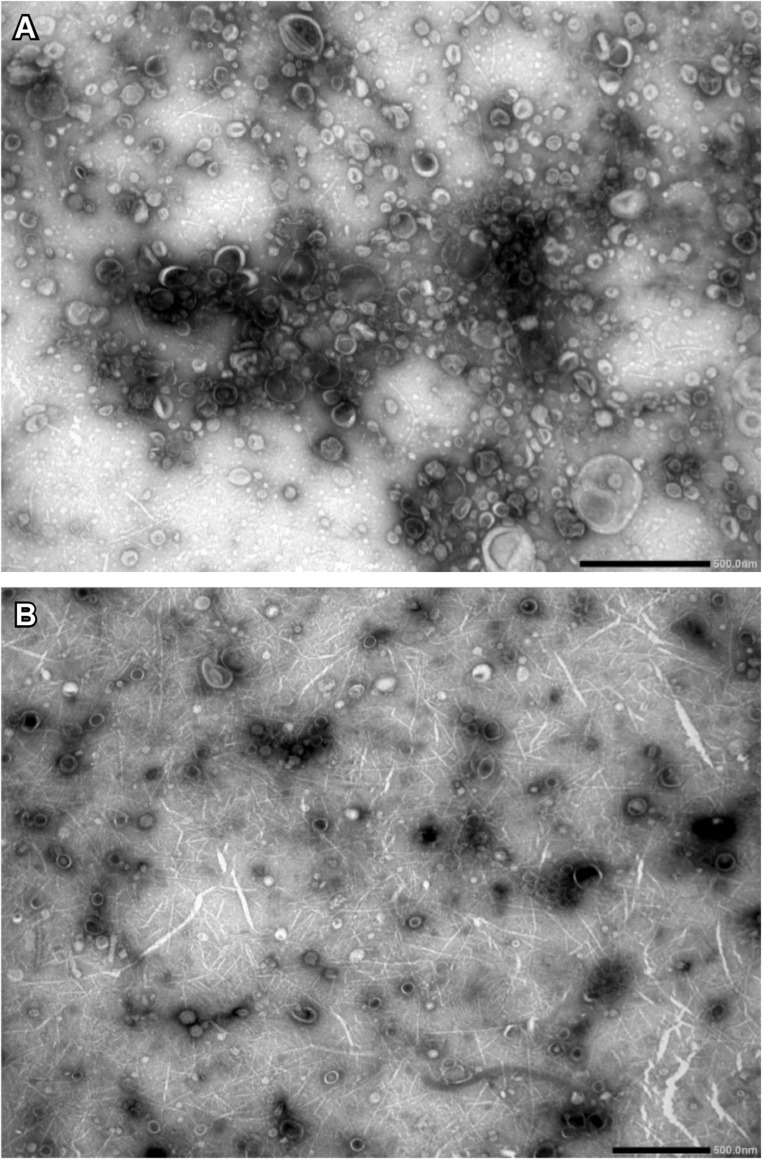
Transmission electron microscopy (TEM) images of exosome/sEV fractions. (**A**) Exosomes/sEVs isolated from tank water. (B) Exosomes/sEVs isolated from morning (AM) open water sample. Spherical vesicles are indicated; vesicles smaller than 50 nm and filamentous structures may correspond to ribosomes and nucleic acids, respectively. Scale bars: 500 nm

Exosomes/sEV fractions were isolated from the high-molecular-weight component of filtered environmental seawater using ultrafiltration. One sample from the rearing tank and one each from the AM and PM aquaculture samples were examined via microscopy. Vesicle size and concentration were measured using interferometric light microscopy (Videodrop; Myriade, Paris, France). The median diameters of the AM and PM samples were 176 nm and 174 nm, respectively (Fig. 3). The concentrations were approximately 5.9 × 10^8^ and 8.0 × 10^8^ vesicles/mL, respectively. These values were similar to those reported for exosomes derived from Akoya pearl oyster tissue and hemolymph in previous studies by Meng et al. (2025) and Huang et al. (2022), as well as to preliminary data from rearing tank water (data not shown). When extrapolated to 1 L of environmental seawater, the estimated vesicle concentration ranged from approximately 1.0 × 10¹⁰ to 10¹¹ vesicles/L. Transmission electron microscopy (TEM) revealed numerous vesicles with diameters primarily in the 50–200 nm range. In addition, vesicles smaller than 50 nm and filamentous structures were observed that were not detectable via interferometric light microscopy (Fig. 4A and B).

Small RNA was extracted from aquaculture water samples collected at the same site in the morning (AM1 and AM2) and in the evening (PM1 and PM2) as well as once from the rearing tank sample. The resulting exosome/sEV preparations were then quantified using the Qubit microRNA assay. Adequate RNA yields were obtained, with concentrations of 9.0 ng/µL for the rearing tank sample and 7.2 ng/µL (AM1), 15.2 ng/µL (AM2), 11.1 ng/µL (PM1), and 20.6 ng/µL (PM2) for the aquaculture samples. Electrophoretic analysis using an Agilent Bioanalyzer showed that the majority of RNA fragments were shorter than 40 nucleotides. In most samples, more than 70% of sequences fell within the characteristic size range for miRNAs.

### Small RNA Profiling of Exosomes/sEVs

Duplicate samples were collected from the aquaculture area (AM and PM), and one sample was collected from the rearing tank. The raw sequencing outputs for the rearing tank and aquaculture samples (AM1, AM2, PM1, and PM2) was 40 million, 30 million, 8 million, 13 million, and 9 million reads (M reads), respectively. After adapter trimming and quality filtering, reads were restricted to a length of 18–40 nucleotides (nt). The remaining read counts were: rearing tank, 15 M reads; AM1, 13 M reads; AM2, 4 M reads; PM1, 6 M reads; PM2, 5 M reads. Mapping these reads to the *P. fucata* reference genome resulted in alignment rates of 4.6% for the tank water sample and 2.8 ± 0.2% for aquaculture area samples. Even though nearly two days elapsed between seawater collection and vesicle isolation, a considerable proportion of reads still mapped to the *P. fucata* reference genome.

**Fig. 5:**
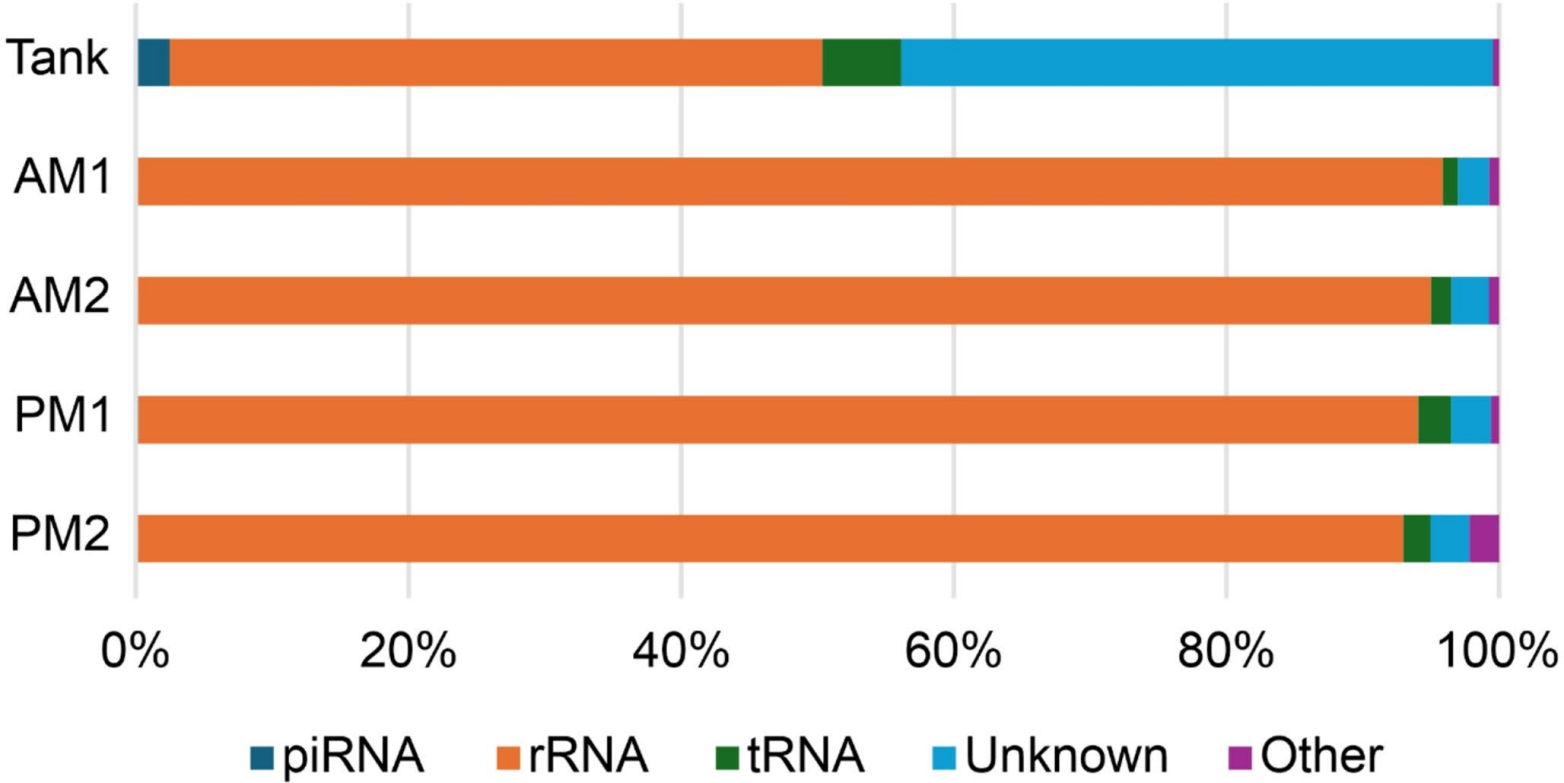
Classifications of mapped small RNA reads from rearing tank and aquaculture water samples. piRNA, piwi-interactive RNA; rRNA, ribosomal RNA; tRNA, transfer RNA; unknown, non-annotated sequence; Other, other small RNAs

Reads were annotated by mapping to the Rfam and miRbase databases. Sequences of 20–24 nt were classified as canonical miRNAs, while sequences of 25–32 nt that did not map to known small RNA categories were designated as estimated piRNAs. Annotated reads were further categorized into piRNA, miRNA, rRNA, tRNA, snRNA, other ncRNAs, and unknown RNA types. Corresponding read counts for each category are presented in Table 1. Among these, rRNA was the most abundant RNA type across all samples (Fig. 5). In contrast, miRNAs accounted for less than 0.1% of mapped reads. Notably, despite being extracted from environmental seawater, piRNAs were detected in higher-than-expected proportions, 2.5% in the rearing tank sample and 0.1 ± 0.06% in aquaculture samples.

**Table 1:** RNA type distribution based on small RNA-seq mapping and annotation

RNA/Reads	Tank	AM1	AM2	PM1	PM2
piRNA	19,248	372	81	246	145
miRNA	27	30	9	22	7
rRNA	373,789	361,592	103,368	171,123	107,696
tRNA	45,384	4,174	1,606	4,321	2,328
snRNA	1,239	1,638	589	717	1,347
other_ncRNAs	2,293	830	173	342	1,088
unknown	338,564	8,702	3,025	5,376	3,306

The sequence most frequently identified among the estimated piRNAs was observed 251 times in the rearing tank samples and 33 times in aquaculture samples (Supplementary data 2). The 1U bias (Kawaoka et al. 2011; Izumi et al. 2013; Stein et al. 2019), calculated based on the number of estimated piRNA sequence types, was 31% in the rearing tank and 22.4 ± 2.1% in the aquaculture area. The corresponding frequency rates were 19.1% and 15.4 ± 5.8%, respectively (Table 2).

**Table 2:** 1U bias based on the number of sequence types and observed frequencies

Sample	Types of sequence			Frequencies of sequence		
	U	A, G, C	1U bias (%)	U	A, G, C	1U bias (%)
Tank	863	2,067	31.3	3,679	15,569	19.1
AM1	71	230	23.6	74	298	19.9
AM2	16	51	23.9	16	65	19.8
PM1	27	112	19.4	35	211	14.2
PM2	8	27	22.9	11	134	7.6

Sequence-type-level analysis indicated that a 1U bias was present only in the rearing tank sample. Comparison of the estimated piRNAs with exosome-derived piRNAs reported by Huang et al. (2022) using BLASTn, revealed that 116 sequence types from the rearing tank and 6 types from the aquaculture area matched exactly with previously reported sequences. Several piRNAs frequently detected in earlier studies were also found in this dataset. Among these, the most abundant piRNA was observed 198 times in the rearing tank sample and 13 times in the aquaculture water sample (Tables 3 and 4; Supplementary data 3).

**Table 3:** Frequently detected piRNA sequences in the rearing tank samples

Top5	Sequence	nt	Number of occurrences in this study	Number of occurrences in previous study*
1	GGAAGGTCCGGAGAGTCTAGGTTACCCAATT	31	198	247
2	GGAAGGTCCGGAGAGTCTAGGTTACCCAATTC	32	171	405
3	TGGGAATACCGGGTGTTGTAGGCAT	25	110	543
4	GGGGTGAGCCGTGGCGTGCTTGGGTTCAAATC	32	79	14
5	ATGGAAGGTCCGGAGAGTCTAGGTTACCCAAT	32	34	336

*: Sequences from the sixth onward are provided in Supplementary data 2. The previous study referenced is Huang et al. (2022)

**Table 4:** Occurrence counts of piRNA sequences matching known sequences in aquaculture water samples

Sample	Sequence	nt	Number of occurrences in this study	Number of occurrences in previous study*
AM1	GATAAATGAAGTCAGCTTATTATCTCTGAGT	31	1	1
	GGCCGTGATCGTATAGTGGTTAGTACTTCGCG	32	1	7,823
	GATTGTCAATGGGTTTTGAGAGAAAACC	28	1	11
	GAGGTCTGCTGCCGATGGACTTTACGATTTCT	32	1	143
	TGTCAATGTACTTTGGATGAGCTGCAGG	28	1	1
PM1	TCAATACCCCTAGCTCAATATTCGACCTTCTC	32	2	3,038
PM2	GGCCGTGATCGTATAGTGGTTAGTACTTCGCG	32	13	7,823

*: The previous study referenced is Huang et al. (2022)

### mRNA Detection from Small RNA-seq Data

mRNA analysis was performed using paired-end reads from the rearing tank and AM1 aquaculture samples, both of which yielded sufficient sequencing depth. After filtering, reads were aligned to the *P. fucata* reference genome. The number of paired-end reads mapped was 56 K reads (Tank) and 120 K reads (AM1). Transcript assembly was performed using the Hisat2–StringTie–Trinotate pipeline. This analysis yielded 164 transcripts from the rearing tank (125 of which were annotated) and 49 transcripts from AM1 (10 annotated), including transcripts lacking gene annotations (Supplementary data 4). Among the detected transcripts were lysosome-associated membrane glycoprotein 1 (LAMP1; Mathieu et al. 2021), moesin (MOES; Li et al. 2024), BRO1 domain-containing protein BROX (BROX; Ichioka et al. 2008), and filamin-A (FLNA; Eguchi et al. 2020), all of which have previously been reported in exosomes/EVs. Additionally, several genes associated with key cellular functions were detected, including those involved in signal transduction (Signal transducer and activator of transcription 5 A; STAT5A), reproduction (Spermatogenesis-associated serine-rich protein 2; SPAS2, Zonadhesin; ZAN), and transport (ATP-binding cassette sub-family A member 2; ABCA2, Sodium- and chloride-dependent glycine transporter 2; SLC6A5).

## Discussion

Exosomes are small extracellular vesicles secreted by eukaryotic cells that contain nucleic acids, proteins, lipids, and other bioactive molecules (Meldolesi 2018; Pegtel and Gould 2019; Kalluri and LeBleu 2020). They have been extensively characterized in bodily fluids and tissues, where they participate in intercellular communication (Gurunathan et al. 2019; O’Brien et al. 2020). However, nearly all prior studies have focused on intracellular or intercellular functions; their release into and potential interaction with environmental seawater remain largely unexplored. Our findings provide the first direct evidence that aquatic organisms, specifically *P. fucata*, release exosomes/sEVs into their surrounding environmental seawater. This observation reveals a previously unrecognized biological phenomenon. We propose that these extra-individual exosomes/sEVs be termed “environmental Exosomes/environmental sEVs (eExosomes/esEVs)”.

By adapting an established exosome/sEV isolation protocol originally developed for cell culture supernatants and liquid biopsy samples (Chen et al. 2022), we successfully obtained vesicle fractions from environmental seawater. These fractions exhibited size distributions consistent with those of tissue- and hemolymph-derived exosomes (Huang et al. 2019, 2022; Meng et al. 2025), as determined by interferometric light microscopy (Videodrop). Transmission electron microscopy further confirmed the presence of abundant vesicles sized 50–200 nm, which aligns with the known size range of exosomes (Huang et al. 2022). These results demonstrate that vesicles morphologically and physically consistent with standard exosomes/sEVs are present in environmental seawater and validate the utility of this modified method for environmental exosome/sEV isolation.

Small RNA-seq of these vesicle fractions revealed that despite a delay of nearly 2 days between sampling and isolation (including storage and transport at 4 °C), a considerable proportion of reads could still be mapped to the *P. fucata* reference genome. While previous studies have shown that naked or free eRNA is highly susceptible to rapid degradation driven by ubiquitous environmental RNases, as well as fluctuations in pH and temperature under such conditions—often declining below the detection threshold within 72 h (Kagzi et al. 2022; Qian et al. 2022; Jo et al. 2023; Wang et al. 2025)—our mapping rate was comparable to or exceeded that reported in conventional eRNA analyses (Veilleux et al. 2021; Miyata et al. 2025). This successful recovery despite the delayed processing strongly supports the hypothesis that aquatic eRNA can exist in a state protected by vesicles (Jo et al. 2022). Specifically, these findings provide compelling evidence that the lipid bilayer of exosomes/sEVs confers robust protection against enzymatic degradation, microbial digestion, and other environmental stressors, thereby preserving the integrity of encapsulated nucleic acids (Koga et al. 2011; Chen et al. 2024). Most of the identified sequences corresponded to rRNA, which may partially reflect the co-isolation of free nucleic acids (Linney et al. 2021) and ribosomes (Maslov et al. 2006) as supported by the presence of filamentous structures and vesicles smaller than 50 nm observed via TEM (Fig. 2). As shown in Tables 2 and 3, we also detected piRNAs with sequences identical to those previously identified in hemolymph-derived exosomes (Huang et al. 2022), providing strong evidence that species-specific small RNAs are released into the environment via exosomes/sEVs.

In the Akoya pearl oyster, piRNAs are not only abundant in reproductive tissues but also enriched in hemolymph-derived exosomes (Huang et al. 2019, 2022). The detection of these piRNAs in environmental seawater strongly supports the analytical capability of our approach to capture species-specific RNA signatures released from the organisms. mRNA analysis using small RNA-seq data identified transcripts for LAMP1 (Mathieu et al. 2021), MOES (Li et al. 2024), BROX (Ichioka et al. 2008), and FLNA (Eguchi et al. 2020), all of which have previously been shown to be associated with exosomes/sEVs. Among these, LAMP1 is considered an exosome-specific surface protein, FLNA is frequently detected in exosomes, and MOES is regarded as a universal pan-exosome marker (Muriel et al. 2016; Hoshino et al. 2020; Mathieu et al. 2021). These findings provide indirect support for the interpretation that the vesicles detected in the environmental seawater samples correspond to exosomes/sEVs. Although their precise association with exosomes/sEVs remains unclear, the presence of transcripts related to reproduction, transport, and signaling may suggest roles in inter-individual communication, such as spawning and horizontal immune transfer. Collectively, our detection of exosome/sEV-encapsulated piRNAs and mRNAs suggests that biological molecules are continuously disseminated beyond individual organisms. While the precise biological functions of these environmental piRNAs and specific mRNAs currently remain uncharacterized and warrant future experimental validation, their stable presence in the water presents significant methodological advantages. Although the specific biological roles of eExosomes/esEVs remain to be determined, our quantification of more than 10 billion vesicles per liter of seawater highlights their potential as a robust source of molecular information. Given that pheromones and other chemical signals exert biological effects at far lower concentrations (Cummins and Bowie 2012), exosomes/sEVs may represent a novel and previously unrecognized mechanism for inter-individual or organismal communication in aquatic ecosystems.

Despite these promising findings, our study has several limitations. First, the exosome/sEV fractions isolated from environmental seawater may include co-isolated particles such as ribosomes and/or free nucleic acids, which could complicate interpretation of the sequencing data. Second, although piRNAs and mRNAs detected in these vesicles suggest potential biological roles, functional validation of their activity in recipient organisms was beyond the scope of this study. Third, our analyses were limited to a single species and sampling period, and additional studies across multiple organisms and environmental contexts will be required to assess the generality of our observations. Addressing these limitations will be essential to fully elucidate the ecological and physiological significance of eExosomes/esEVs.

In conclusion, this study demonstrates for the first time that aquatic organisms release exosomes/sEVs containing species-specific nucleic acids into their surrounding waters. By stabilizing and transmitting functional molecules, eExosomes/esEVs may represent a previously unrecognized mode of inter-organismal communication and contribute to the formation of complex environmental information networks. Notably, unlike eRNA, which rapidly degrades under natural conditions, eExosomes/esEVs provide a stable source of molecular information, suggesting that they may complement existing eDNA and eRNA approaches in ecological monitoring. Future research should aim to elucidate the mechanisms, dynamics, and ecological significance of eExosomes/esEVs across diverse aquatic organisms.

## Supplementary Information

Below is the link to the electronic supplementary material.

Supplementary Material 1 (XLSX 51.5 KB)

Supplementary Material 2 (XLSX 19.0 KB)

Supplementary Material 3 (XLSX 122 KB)

Supplementary Material 4 (TXT 4.00 KB)

Supplementary Material 5 (PDF 120 KB)

## Data Availability

The NGS data have been deposited with links to BioProject accession number PRJDB37606 in the DNA Data Bank of Japan (DDBJ) BioProject database. Other data generated or analyzed during this study are included in this article and supplementary information.

## References

[CR1] Aminaka Y, Wong M-S, Yada T, Hyodo S (2025) The use of environmental RNA for inferring fish spawning behavior. Sci Rep 15:37559. 10.1038/s41598-025-23861-8 41198842 10.1038/s41598-025-23861-8 PMC12592704

[CR2] Beier S, Thiel T, Münch T, Scholz U, Mascher M (2017) MISA-web: a web server for microsatellite prediction. Bioinformatics 33:2583–2585. 10.1093/bioinformatics/btx198 28398459 10.1093/bioinformatics/btx198 PMC5870701

[CR3] Benson G (1999) Tandem repeats finder: a program to analyze DNA sequences. Nucleic Acids Res 27:573–580. 10.1093/nar/27.2.573 9862982 10.1093/nar/27.2.573 PMC148217

[CR4] Bryant DM, Johnson K, DiTommaso T, Tickle T, Couger MB, Payzin-Dogru D, Lee TJ, Leigh ND, Kuo T-H, Davis FG, Bateman J, Bryant S, Guzikowski AR, Tsai SL, Coyne S, Ye WW, Freeman RM, Peshkin L, Tabin CJ, Regev A, Haas BJ, Whited JL (2017) A tissue-mapped axolotl de novo transcriptome enables identification of limb regeneration factors. Cell Rep 18:762–776. 10.1016/j.celrep.2016.12.063 28099853 10.1016/j.celrep.2016.12.063 PMC5419050

[CR5] Bunholi IV, Foster NR, Casey JM (2023) Environmental DNA and RNA in aquatic community ecology: toward methodological standardization. Environ DNA 5:1133–1147. 10.1002/edn3.476

[CR6] Chen H, Li Q (2025) Recent advances in scalable exosome production: challenges and innovations. Chin J Plast Reconstr Surg. 10.1016/j.cjprs.2025.05.001

[CR7] Chen J, Li P, Zhang T, Xu Z, Huang X, Wang R, Du L (2022) Review on strategies and technologies for exosome isolation and purification. Front Bioeng Biotechnol 9:811971. 10.3389/fbioe.2021.811971 35071216 10.3389/fbioe.2021.811971 PMC8766409

[CR8] Chen S, Zhou Y, Chen Y, Gu J (2018) Fastp: an ultra-fast all-in-one FASTQ preprocessor. Bioinformatics 34:i884–i890. 10.1093/bioinformatics/bty560 30423086 10.1093/bioinformatics/bty560 PMC6129281

[CR9] Chen Y-F, Luh F, Ho Y-S, Yen Y (2024) Exosomes: a review of biologic function, diagnostic and targeted therapy applications, and clinical trials. J Biomed Sci 31:67. 10.1186/s12929-024-01055-0 38992695 10.1186/s12929-024-01055-0 PMC11238361

[CR10] Cummins SF, Bowie JH (2012) Pheromones, attractants and other chemical cues of aquatic organisms and amphibians. Nat Prod Rep 29:642–658. 10.1039/c2np00102k 22495567 10.1039/c2np00102k

[CR11] Dad HA, Gu T-W, Zhu A-Q, Huang L-Q, Peng L-H (2021) Plant Exosome-like Nanovesicles: Emerging Therapeutics and Drug Delivery Nanoplatforms. Mol Ther 29:13–31. 10.1016/j.ymthe.2020.11.030 33278566 10.1016/j.ymthe.2020.11.030 PMC7791080

[CR12] Eguchi A, Fukuda S, Kuratsune H, Nojima J, Nakatomi Y, Watanabe Y, Feldstein AE (2020) Identification of actin network proteins, talin-1 and filamin-A, in circulating extracellular vesicles as blood biomarkers for human myalgic encephalomyelitis/chronic fatigue syndrome. Brain Behav Immun 84:106–114. 10.1016/j.bbi.2019.11.015 31759091 10.1016/j.bbi.2019.11.015 PMC7010541

[CR13] Gabaran SG, Nejati V, Dilsiz N, Rezaie J (2025) An updated review on the inhibition of exosome biogenesis, release, and uptake: a potential anticancer approach. Biochem Pharmacol 239:117019. 10.1016/j.bcp.2025.117019 40499840 10.1016/j.bcp.2025.117019

[CR14] Glover CN, Veilleux HD, Misutka MD (2025) Commentary: Environmental RNA and the assessment of organismal function in the field. Comp Biochem Physiol B Biochem Mol Biol 275:111036. 10.1016/j.cbpb.2024.111036 39313021 10.1016/j.cbpb.2024.111036

[CR15] Gurunathan S, Kang M-H, Jeyaraj M, Qasim M, Kim J-H (2019) Review of the Isolation, Characterization, Biological Function, and Multifarious Therapeutic Approaches of Exosomes. Cells 8:307. 10.3390/cells8040307 30987213 10.3390/cells8040307 PMC6523673

[CR16] Gurung S, Perocheau D, Touramanidou L, Baruteau J (2021) The exosome journey: from biogenesis to uptake and intracellular signalling. Cell Commun Signal 19:47. 10.1186/s12964-021-00730-1 33892745 10.1186/s12964-021-00730-1 PMC8063428

[CR17] Haas BJ, Papanicolaou A, Yassour M, Grabherr M, Blood PD, Bowden J, Couger MB, Eccles D, Li B, Lieber M, MacManes MD, Ott M, Orvis J, Pochet N, Strozzi F, Weeks N, Westerman R, William T, Dewey CN, Henschel R, LeDuc RD, Friedman N, Regev A (2013) De novo transcript sequence reconstruction from RNA-seq using the Trinity platform for reference generation and analysis. Nat Protoc 8:1494–1512. 10.1038/nprot.2013.084 23845962 10.1038/nprot.2013.084 PMC3875132

[CR18] Hechler RM, Yates MC, Chain FJJ, Cristescu ME (2025) Environmental transcriptomics under heat stress: can environmental RNA reveal changes in gene expression of aquatic organisms? Mol Ecol 34:e17152. 10.1111/mec.17152 37792902 10.1111/mec.17152 PMC12186723

[CR19] Hiki K, Jo TS (2025) Comprehensive sequencing of environmental RNA from Japanese medaka at various size fractions and comparison with skin swab RNA. Environ DNA. 10.1002/edn3.70137

[CR20] Hiki K, Yamagishi T, Yamamoto H (2023) Environmental RNA as a noninvasive tool for assessing toxic effects in fish: a proof-of-concept study using Japanese medaka exposed to pyrene. Environ Sci Technol 57:12654–12662. 10.1021/acs.est.3c03737 37585234 10.1021/acs.est.3c03737

[CR21] Hoshino A, Kim HS, Bojmar L, Gyan KE, Cioffi M, Hernandez J, Zambirinis CP, Rodrigues G, Molina H, Heissel S, Mark MT, Steiner L, Benito-Martin A, Lucotti S, Giannatale AD, Offer K, Nakajima M, Williams C, Nogués L, Vatter FAP, Hashimoto A, Davies AE, Freitas D, Kenific CM, Ararso Y, Buehring W, Lauritzen P, Ogitani Y, Sugiura K, Takahashi N, Alečković M, Bailey KA, Jolissant JS, Wang H, Harris A, Schaeffer LM, García-Santos G, Posner Z, Balachandran VP, Khakoo Y, Raju GP, Scherz A, Sagi I, Scherz-Shouval R, Yarden Y, Oren M, Malladi M, Petriccione M, Braganca KCD, Donzelli M, Fischer C, Vitolano S, Wright GP, Ganshaw L, Marrano M, Ahmed A, DeStefano J, Danzer E, Roehrl MHA, Lacayo NJ, Vincent TC, Weiser MR, Brady MS, Meyers PA, Wexler LH, Ambati SR, Chou AJ, Slotkin EK, Modak S, Roberts SS, Basu EM, Diolaiti D, Krantz BA, Cardoso F, Simpson AL, Berger M, Rudin CM, Simeone DM, Jain M, Ghajar CM, Batra SK, Stanger BZ, Bui J, Brown KA, Rajasekhar VK, Healey JH, Sousa M, Kramer K, Sheth S, Baisch J, Pascual V, Heaton TE, Quaglia MPL, Pisapia DJ, Schwartz R, Zhang H, Liu Y, Shukla A, Blavier L, DeClerck YA, LaBarge M, Bissell MJ, Caffrey TC, Grandgenett PM, Hollingsworth MA, Bromberg J, Costa-Silva B, Peinado H, Kang Y, Garcia BA, O’Reilly EM, Kelsen D, Trippett TM, Jones DR, Matei IR, Jarnagin WR, Lyden D (2020) Extracellular vesicle and particle biomarkers define multiple human cancers. Cell 182:1044-1061e.e18. 10.1016/j.cell.2020.07.009 32795414 10.1016/j.cell.2020.07.009 PMC7522766

[CR22] Huang S, Ichikawa Y, Igarashi Y, Yoshitake K, Kinoshita S, Omori F, Maeyama K, Nagai K, Watabe S, Asakawa S (2019) Piwi-interacting RNA (piRNA) expression patterns in pearl oyster (*Pinctada fucata*) somatic tissues. Sci Rep 9:247. 10.1038/s41598-018-36726-0 30670741 10.1038/s41598-018-36726-0 PMC6342924

[CR23] Huang S, Nishiumi S, Asaduzzaman M, Pan Y, Liu G, Yoshitake K, Maeyama K, Kinoshita S, Nagai K, Watabe S, Yoshida T, Asakawa S (2022) Exosome-derived small non-coding RNAs reveal immune response upon grafting transplantation in *Pinctada fucata* (Mollusca). Open Biol 12:210317. 10.1098/rsob.210317 35506205 10.1098/rsob.210317 PMC9065966

[CR24] Huang S, Yoshitake K, Asakawa S (2021) A review of discovery profiling of PIWI-interacting RNAs and their diverse functions in metazoans. Int J Mol Sci 22:11166. 10.3390/ijms222011166 34681826 10.3390/ijms222011166 PMC8538981

[CR25] Ichioka F, Kobayashi R, Katoh K, Shibata H, Maki M (2008) Brox, a novel farnesylated Bro1 domain-containing protein that associates with charged multivesicular body protein 4 (CHMP4). FEBS J 275:682–692. 10.1111/j.1742-4658.2007.06230.x 18190528 10.1111/j.1742-4658.2007.06230.x

[CR26] Izumi N, Kawaoka S, Yasuhara S, Suzuki Y, Sugano S, Katsuma S, Tomari Y (2013) Hsp90 facilitates accurate loading of precursor piRNAs into PIWI proteins. RNA 19:896–901. 10.1261/rna.037200.112 23681506 10.1261/rna.037200.112 PMC3683924

[CR27] Jo T, Tsuri K, Hirohara T, Yamanaka H (2023) Warm temperature and alkaline conditions accelerate environmental RNA degradation. Environ DNA 5:836–848. 10.1002/edn3.334

[CR28] Jo TS, Matsuda N, Hirohara T, Yamanaka H (2022) Simple and efficient preservation of fish environmental RNA in filtered water samples via RNAlater. Preprint at Research Square. 10.21203/rs.3.rs-2170577/v1.

[CR29] Kagzi K, Hechler RM, Fussmann GF, Cristescu ME (2022) Environmental RNA degrades more rapidly than environmental DNA across a broad range of pH conditions. Mol Ecol Resour 22:2640–2650. 10.1111/1755-0998.13655 35643953 10.1111/1755-0998.13655

[CR30] Kalluri R, LeBleu VS (2020) The biology, function, and biomedical applications of exosomes. Science. 10.1126/science.aau6977 32029601 10.1126/science.aau6977 PMC7717626

[CR31] Kalvari I, Nawrocki EP, Ontiveros-Palacios N, Argasinska J, Lamkiewicz K, Marz M, Griffiths-Jones S, Toffano-Nioche C, Gautheret D, Weinberg Z, Rivas E, Eddy SR, Finn RD, Bateman A, Petrov AI (2020) Rfam 14: expanded coverage of metagenomic, viral and microRNA families. Nucleic Acids Res 49:D192–D200. 10.1093/nar/gkaa1047 10.1093/nar/gkaa1047 PMC7779021 33211869

[CR32] Kawaoka S, Izumi N, Katsuma S, Tomari Y (2011) 3′ end formation of PIWI-interacting RNAs in vitro. Mol Cell 43:1015–1022. 10.1016/j.molcel.2011.07.029 21925389 10.1016/j.molcel.2011.07.029

[CR33] Kim D, Paggi JM, Park C, Bennett C, Salzberg SL (2019) Graph-based genome alignment and genotyping with HISAT2 and HISAT-genotype. Nat Biotechnol 37:907–915. 10.1038/s41587-019-0201-4 31375807 10.1038/s41587-019-0201-4 PMC7605509

[CR34] Kim HG, Kwon K, Suh H, Lee S, Park K, Kwon O, Choi J (2015) Exosome isolation from hemolymph of Korean rhinoceros beetle, *Allomyrina dichotoma* (Coleoptera: Scarabaeidae). Entomol Res 45:339–344. 10.1111/1748-5967.12140

[CR35] Koga Y, Yasunaga M, Moriya Y, Akasu T, Fujita S, Yamamoto S, Matsumura Y (2011) Exosome can prevent RNase from degrading microrna in feces. J Gastrointest Oncol 2:215–222. 10.3978/j.issn.2078-6891.2011.015 22811855 10.3978/j.issn.2078-6891.2011.015 PMC3397623

[CR36] Kozomara A, Birgaoanu M, Griffiths-Jones S (2019) miRBase: from microrna sequences to function. Nucleic Acids Res 47:D155–D162. 10.1093/nar/gky1141 30423142 10.1093/nar/gky1141 PMC6323917

[CR37] Langmead B, Trapnell C, Pop M, Salzberg SL (2009) Ultrafast and memory-efficient alignment of short DNA sequences to the human genome. Genome Biol 10:R25. 10.1186/gb-2009-10-3-r25 19261174 10.1186/gb-2009-10-3-r25 PMC2690996

[CR38] Li B, Kugeratski FG, Kalluri R (2024) A novel machine learning algorithm selects proteome signature to specifically identify cancer exosomes. Elife 12:RP90390. 10.7554/elife.90390 38529947 10.7554/eLife.90390 PMC10965221

[CR39] Li H (2013) Aligning sequence reads, clone sequences and assembly contigs with BWA-MEM. arXiv. 10.48550/arxiv.1303.3997

[CR40] Li H, Handsaker B, Wysoker A, Fennell T, Ruan J, Homer N, Marth G, Abecasis G, Durbin R, Subgroup 1000 Genome Project Data Processing (2009) The sequence alignment/map format and SAMtools. Bioinformatics 25:2078–2079. 10.1093/bioinformatics/btp352 19505943 10.1093/bioinformatics/btp352 PMC2723002

[CR41] Linney MD, Schvarcz CR, Steward GF, DeLong EF, Karl DM (2021) A method for characterizing dissolved DNA and its application to the North Pacific Subtropical Gyre. Limnol Oceanogr Methods 19:210–221. 10.1002/lom3.10415

[CR42] Maslov DA, Sharma MR, Butler E, Falick AM, Gingery M, Agrawal RK, Spremulli LL, Simpson L (2006) Isolation and characterization of mitochondrial ribosomes and ribosomal subunits from *Leishmania tarentolae*. Mol Biochem Parasitol 148:69–78. 10.1016/j.molbiopara.2006.02.021 16600399 10.1016/j.molbiopara.2006.02.021

[CR43] Mathieu M, Névo N, Jouve M, Valenzuela JI, Maurin M, Verweij FJ, Palmulli R, Lankar D, Dingli F, Loew D, Rubinstein E, Boncompain G, Perez F, Théry C (2021) Specificities of exosome versus small ectosome secretion revealed by live intracellular tracking of CD63 and CD9. Nat Commun 12:4389. 10.1038/s41467-021-24384-2 34282141 10.1038/s41467-021-24384-2 PMC8289845

[CR44] Meldolesi J (2018) Exosomes and ectosomes in intercellular communication. Curr Biol 28:R435–R444. 10.1016/j.cub.2018.01.059 29689228 10.1016/j.cub.2018.01.059

[CR45] Meng L, Pan Y, Yonezawa R, Yang K, Bailey-Kobayashi N, Hashimoto N, Maeyama K, Yoshitake K, Kinoshita S, Yoshida T, Nagai K, Watabe S, Asakawa S (2025) Identification and comparison of exosomal and non-exosomal microRNAs in mantle tissue of *Pinctada fucata* (Akoya pearl oyster). Int J Biol Macromol 309:142991. 10.1016/j.ijbiomac.2025.142991 40210052 10.1016/j.ijbiomac.2025.142991

[CR46] Minamoto T, Miya M, Sado T, Seino S, Doi H, Kondoh M, Nakamura K, Takahara T, Yamamoto S, Yamanaka H, Araki H, Iwasaki W, Kasai A, Masuda R, Uchii K (2021) An illustrated manual for environmental DNA research: water sampling guidelines and experimental protocols. Environ DNA 3:8–13. 10.1002/edn3.121

[CR47] Miyata K, Inoue Y, Amano Y, Nishioka T, Yamane M, Kawaguchi T, Morita O, Honda H (2021) Fish environmental RNA enables precise ecological surveys with high positive predictivity. Ecol Indic 128:107796. 10.1016/j.ecolind.2021.107796

[CR48] Miyata K, Inoue Y, Yamane M, Honda H (2025) Fish environmental RNA sequencing sensitively captures accumulative stress responses through short-term aquarium sampling. Sci Total Environ 959:178182. 10.1016/j.scitotenv.2024.178182 39719761 10.1016/j.scitotenv.2024.178182

[CR49] Moros M, Fergola E, Marchesano V, Mutarelli M, Tommasini G, Miedziak B, Palumbo G, Ambrosone A, Tino A, Tortiglione C (2021) The aquatic invertebrate Hydra vulgaris releases molecular messages through extracellular vesicles. Front Cell Dev Biol 9:788117. 10.3389/fcell.2021.788117 34988080 10.3389/fcell.2021.788117 PMC8721104

[CR50] Muriel O, Tomas A, Scott CC, Gruenberg J (2016) Moesin and cortactin control actin-dependent multivesicular endosome biogenesis. Mol Biol Cell 27:3305–3316. 10.1091/mbc.e15-12-0853 27605702 10.1091/mbc.E15-12-0853 PMC5170863

[CR51] Nazri HM, Greaves E, Quenby S, Dragovic R, Tapmeier TT, Becker CM (2023) The role of small extracellular vesicle-miRNAs in endometriosis. Hum Reprod 38:2296–2311. 10.1093/humrep/dead216 37877421 10.1093/humrep/dead216 PMC10694411

[CR52] Negahdaripour M, Owji H, Eskandari S, Zamani M, Vakili B, Nezafat N (2021) Small extracellular vesicles (sEVs): discovery, functions, applications, detection methods and various engineered forms. Expert Opin Biol Ther 21:371–394. 10.1080/14712598.2021.1825677 32945228 10.1080/14712598.2021.1825677

[CR53] O’Brien K, Breyne K, Ughetto S, Laurent LC, Breakefield XO (2020) RNA delivery by extracellular vesicles in mammalian cells and its applications. Nat Rev Mol Cell Biol 21:585–606. 10.1038/s41580-020-0251-y 32457507 10.1038/s41580-020-0251-y PMC7249041

[CR54] Pan Y, Meng L, Yoshida K, Qiu L, Ito T, Yonezawa R, Yoshitake K, Saito S, Bailey-Kobayashi N, Yoshida T, Kinoshita S, Asakawa S (2025) Comparative and functional analysis of exosomal microRNAs during semelparous reproduction in Ayu fish (*Plecoglossus altivelis*). J Extracell Biol 4:e70038. 10.1002/jex2.70038 40066203 10.1002/jex2.70038 PMC11891394

[CR55] Pegtel DM, Gould SJ (2019) Exosomes. Annu Rev Biochem 88:487–514. 10.1146/annurev-biochem-013118-111902 31220978 10.1146/annurev-biochem-013118-111902

[CR56] Pertea M, Pertea GM, Antonescu CM, Chang T-C, Mendell JT, Salzberg SL (2015) StringTie enables improved reconstruction of a transcriptome from RNA-seq reads. Nat Biotechnol 33:290–295. 10.1038/nbt.3122 25690850 10.1038/nbt.3122 PMC4643835

[CR57] Qian T, Shan X, Wang W, Jin X (2022) Effects of temperature on the timeliness of eDNA/eRNA: a case study of *Fenneropenaeus chinensis*. Water 14:1155. 10.3390/w14071155

[CR58] Sausset R, Krupova Z, Guédon E, Peron S, Grangier A, Petit M, Sordi LD, Paepe MD (2023) Comparison of interferometric light microscopy with nanoparticle tracking analysis for the study of extracellular vesicles and bacteriophages. J Extracell Biol 2:e75. 10.1002/jex2.75 38938523 10.1002/jex2.75 PMC11080698

[CR59] Shen W, Le S, Li Y, Hu F (2016) SeqKit: a cross-platform and ultrafast toolkit for FASTA/Q file manipulation. PLoS ONE 11:e0163962. 10.1371/journal.pone.0163962 27706213 10.1371/journal.pone.0163962 PMC5051824

[CR60] Shi L, Guo C, Fang M, Yang Y, Yin F, Shen Y (2024) Cross-kingdom regulation of plant microRNAs: potential application in crop improvement and human disease therapeutics. Front Plant Sci 15:1512047. 10.3389/fpls.2024.1512047 39741676 10.3389/fpls.2024.1512047 PMC11685121

[CR61] Stein CB, Genzor P, Mitra S, Elchert AR, Ipsaro JJ, Benner L, Sobti S, Su Y, Hammell M, Joshua-Tor L, Haase AD (2019) Decoding the 5′ nucleotide bias of PIWI-interacting RNAs. Nat Commun 10:828. 10.1038/s41467-019-08803-z 30783109 10.1038/s41467-019-08803-z PMC6381166

[CR62] Takeuchi T, Suzuki Y, Watabe S, Nagai K, Masaoka T, Fujie M, Kawamitsu M, Satoh N, Myers EW (2022) A high-quality, haplotype-phased genome reconstruction reveals unexpected haplotype diversity in a pearl oyster. DNA Res 29:dsac035. 10.1093/dnares/dsac035 36351462 10.1093/dnares/dsac035 PMC9646362

[CR63] Valadi H, Ekström K, Bossios A, Sjöstrand M, Lee JJ, Lötvall JO (2007) Exosome-mediated transfer of mRNAs and microRNAs is a novel mechanism of genetic exchange between cells. Nat Cell Biol 9:654–659. 10.1038/ncb1596 17486113 10.1038/ncb1596

[CR64] Veilleux HD, Misutka MD, Glover CN (2021) Environmental DNA and environmental RNA: current and prospective applications for biological monitoring. Sci Total Environ 782:146891. 10.1016/j.scitotenv.2021.146891 33848866 10.1016/j.scitotenv.2021.146891

[CR65] Wang F, Xiong W, Huang X, Zhan A (2025) Influence of short-term water sample storage on environmental RNA metabarcoding-based biodiversity assessment. J Environ Sci. 10.1016/j.jes.2025.11.019 10.1016/j.jes.2025.11.019 42336528

[CR66] Ważny Ł, Whiteside TL, Pietrowska M (2024) Oncoviral infections and small extracellular vesicles. Viruses 16:1291. 10.3390/v16081291 39205265 10.3390/v16081291 PMC11359865

[CR67] Yates MC, Derry AM, Cristescu ME (2021) Environmental RNA: a revolution in ecological resolution? Trends Ecol Evol 36:601–609. 10.1016/j.tree.2021.03.001 33757695 10.1016/j.tree.2021.03.001

[CR68] Zhang L, Liu X, Wang C, Li X, Tian G, Wang W (2023) Cell-Free RNA Sequencing from Biofluid Samples. In: Huang T, Yang J, Tian G (eds) Liquid Biopsies, Methods and Protocols. Humana, pp 9–26

[CR69] Zhao F, Cheng L, Shao Q, Chen Z, Lv X, Li J, He L, Sun Y, Ji Q, Lu P, Ji Y, Ji J (2020) Characterization of serum small extracellular vesicles and their small RNA contents across humans, rats, and mice. Sci Rep 10:4197. 10.1038/s41598-020-61098-9 32144372 10.1038/s41598-020-61098-9 PMC7060188

[CR70] Zheng Z, Xu Z, Cai C, Liao Y, Yang C, Du X, Huang R, Deng Y (2022) Circulating exosome miRNA, is it the novel nutrient molecule through cross-kingdom regulation mediated by food chain transmission from microalgae to bivalve? Comp Biochem Physiol Part D Genomics Proteomics 43:101004. 10.1016/j.cbd.2022.101004 35644102 10.1016/j.cbd.2022.101004

[CR71] Zhou X, Huang J, Zhang D, Qian Z, Zuo X, Sun Y (2025) Small extracellular vesicles: the origins, current status, future prospects, and applications. Stem Cell Res Ther 16:184. 10.1186/s13287-025-04330-5 40247402 10.1186/s13287-025-04330-5 PMC12004682

