# Supplementary material for: Environmental Exosomes/Small Extracellular Vesicles: Evidence of Extracellular RNA Release by Aquatic Organisms
Source: Mar Biotechnol (NY). 2026 Apr 25;28(3):72. doi: 10.1007/s10126-026-10618-1 (PMC13110240; doi:10.1007/s10126-026-10618-1)
Supplement: Supplementary file 5 — Supplementary Material 5 (PDF 120 KB) [file 10126_2026_10618_MOESM5_ESM.pdf]

Supplementary Information

Environmental exosomes/small extracellular vesicles: Evidence of extracellular RNA release by aquatic organisms

Authors

Ryo Yonezawa [1, 2, 5*], Lingxin Meng [1], Naoki Hashimoto [8], Ibuki Igarashi[3], Satoshi Kimura[4], Nina Yasuda[3], Susumu Mitsuyama [1, 2], Takanori Kobayashi [1, 2], Kazutoshi Yoshitake [2, 6], Shigeharu Kinoshita [1], Nahoko Bailey-Kobayashi [7], Kaoru Maeyama [9], Kiyohito Nagai [8], Shugo Watabe [6], Tetsuhiko Yoshida [7], Shuichi Asakawa [1, 2*]

[1] Laboratory of Aquatic Molecular Biology and Biotechnology, Department of Aquatic Bioscience, Graduate School of Agricultural and Life Science, The University of Tokyo, Bunkyo, Tokyo 113-8657, Japan
[2] Signal Peptidome Research Laboratory, Department of Aquatic Bioscience, Graduate School of Agricultural and Life Sciences, The University of Tokyo, Bunkyo, Tokyo 113-8657, Japan
[3] Laboratory of Aquatic Conservation, Department of Ecosystem Studies, Graduate School of Agricultural and Life Science, The University of Tokyo, Bunkyo, Tokyo 113-8657, Japan
[4] Technology Advancement Center, Graduate School of Agricultural and Life Sciences, The University of Tokyo, Bunkyo-ku, Tokyo 113-8657, Japan
[5] College of Bioresource Sciences, Nihon University, Kanagawa, 252-0880, Japan
[6] School of Marine Biosciences, Kitasato University, Minami-ku, Sagamihara, Kanagawa 252-0313, Japan
[7] Institute for Advanced Sciences, TOAGOSEI CO., LTD., Tsukuba, Ibaraki 300-2611, Japan
[8] Mikimoto Pearl Research Institute, K.MIKIMOTO & CO., LTD., Hazako 923-74, Hamajima, Shima, Mie 517-0403, Japan
[9] Mikimoto Pharmaceutical CO., LTD., Kurose 1425, Ise, Mie 516-8581, Japan

*Corresponding authors:
Ryo Yonezawa ; E-mail: ryonezawa@g.ecc.u-tokyo.ac.jp, yonezawa.ryo@nihon-u.ac.jp
Shuichi Asakawa; E-mail: asakawa@g.ecc.u-tokyo.ac.jp

Data legends

Supplementary data S1. (separate file)
Custom Perl script (classify_rna.pl) used to classify RNA types and generate corresponding FASTA sequence files.
Note: This file is named "Supplementary_file_1.txt"; please rename it to "classify_rna.pl" before use.

Supplementary data S2. (separate file)
Complete list of estimated piRNA sequences and their read counts detected in the rearing tank and aquaculture samples.

Supplementary data S3. (separate file)
Complete list of occurrence counts of piRNA sequences matching known sequences in rearing tank sample.

Supplementary data S4 (separate file)
Summary of transcript assembly and annotation results generated by the Hisat2–StringTie–Trinotate pipeline, including transcript IDs, sample origin (Rearing tank/aquaculture; AM1), and BLASTx/BLASTp annotation data.
